# Supplementary material for: Learning curve and surgical outcome of robotic assisted colorectal surgery with ERAS program
Source: Sci Rep. 2022 Nov 29;12:20566. doi: 10.1038/s41598-022-24665-w (PMC9709162; doi:10.1038/s41598-022-24665-w)
Supplement: Supplementary file 2 — Supplementary Information 2. [file 41598_2022_24665_MOESM2_ESM.zip › RAL Raw data-Ñ[▒K/108/OP_ñw╝╨░O▒Kñσ.pdf]

正式摘要(電子簽章：0000002236835847)

W52 / W52

臺中榮民總醫院

Operation Note

[redacted]

- 手術日期 : 2021/08/25
- 手術醫師 : VS 林俊余 , CR 楊曙榮 , R 郭連雄
- Anesthesia : GE
- Position : Lithotomy
- Aseptic agents : Soap , Betadine Alcohol , Betadine Aqua

Diagnosis

- Pre-operative diagnosis : S COLON CA.
- Post-operative diagnosis : Ditto

Surgical Indication

- Tumor resection

Operation

- Robot Assisted Colectomy(L)

Finding

- 1. One 3x4 cm tumor located at middle S-colon. cutend: proximal 10 cm, distal 10 cm.
  2. There was no palpable liver metastasis, no visible peritoneal seeding.
  3. Adhesion of small bowel loop on mesentary and s/p lysis by robot.
  4. High ligation (+) above the left colic artery.
     Take down spelnic flexure +
  5. No Drain. Hyalobarrer +
  6. ICG(25mg/10ml) perfusion: Rectum: 3ml, 30sec. Colon: 3ml, 40sec, Level Correction:+

Procedure

- Under GE, the patient was put in lithotomy position. 5 troca hole was made, the first was by open method. Pneumoperitonium was created. the robotic endoscope was inserted. Abdominal exploration was done and the findings were mentioned above. The patient position was adjusted to right tilting to let small bowels move to Rt side and Lt side colon was well exposured. The IMA was identified, dissected and transected by hemolock. The IMV was done in the same way. The mobilization of S-colon was done with dissection via embryonic plane between Gerota fascia and meso-sigmoid colon. The lateral attachement of sigmoid colon was also divided. After fully mobilization of S-colon, the rectum was also dissected posteriorly in upper rectum level, Denovillier fascia anteriorly. The bilateral lateral ligment was divided. Transection of rectum at peritoneal reflection was done by two 45 mm endocutters. Then the splenic flexure was taken down. The speciemn was removed from LLQ wound . The 29 mm envil was inserted in proximal colon annd take back into abdominal cavity. The end to end colorectal anastomosis was done by double stappling technique. After air-leak test, penrosce drain was placed to the pelvic from RLQ. 5 ports and was closed in layers. Throughout the whole course of operation, patient stood quite well.
- Estimated blood loss: minimum
- 器械數量: 正確
- 紗布數量: 正確

Image

- PAINT
  - abdomen

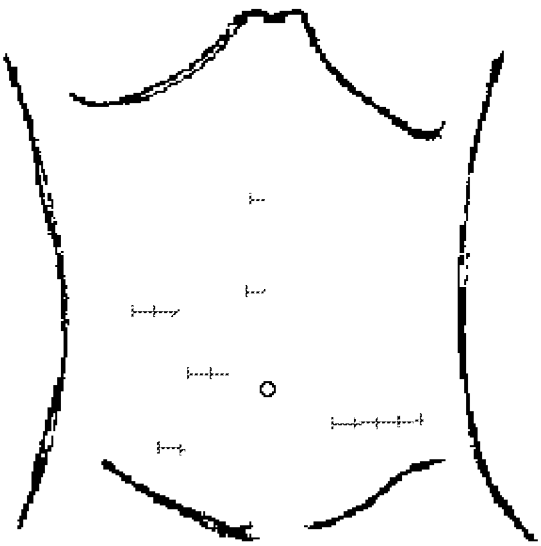

  - colon & rectum

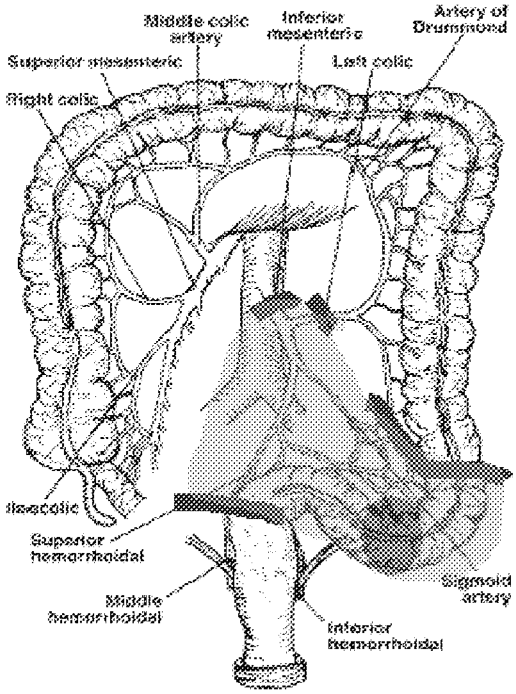

主治 2222H林俊余 / 醫師 2292G楊曙榮

正式摘要(電子簽章：**000000223685847)**

W52 / W52

doctorRank:B
